# Cross‐stressor resilience of soil microbial growth and carbon metabolism under climate change

**DOI:** 10.1002/ecy.70439

**Published:** 2026-06-21

**Authors:** Jin‐Tao Lí, Lettice C. Hicks, Albert C. Brangarí, Johannes Rousk

**Affiliations:** ^1^ Microbial Biogeochemistry in Lund (MBLU), Department of Biology Lund University Lund Sweden; ^2^ State Key Laboratory of Wetland Conservation and Restoration, National Observations and Research Station for Wetland Ecosystems of the Yangtze Estuary Ministry of Education Key Laboratory for Biodiversity Science and Ecological Engineering, and Institute of Eco‐Chongming, School of Life Sciences, Fudan University Shanghai China; ^3^ Microbial Ecology, Department of Biology Lund University Lund Sweden; ^4^ Department of Ecosystem and Landscape Dynamics Institute for Biodiversity and Ecosystem Dynamics, University of Amsterdam Amsterdam the Netherlands; ^5^ Department of Physical Geography and Ecosystem Science Lund University Lund Sweden

**Keywords:** disturbance ecology, drying‐rewetting, ecosystem functional stability, freeze–thaw, global change ecology, microbial growth efficiency, tolerance

## Abstract

The microbial ability to recover metabolism after perturbation events ensures ecosystem functional stability in a changing climate, where multiple climatic stressors increasingly occur in sequential and seasonally cyclic patterns. While prior exposure to a specific stress can enhance microbial resilience to that stress, whether this resilience extends to different stressors remains largely unexplored. Here, we investigated cross‐stressor resilience of microbial communities by testing how prior exposure to one type of perturbation (frost or drought) affects microbial resilience to subsequent perturbations of either type in soil systems. We found that prior exposure to drought or frost enhanced the resilience of microbial growth to subsequent perturbations of either type and enabled the maintenance of higher microbial carbon use efficiency. It is likely that this cross‐stressor resilience arose because frost and drought both can exert stress on microbes via effects on water potential. This suggests that induced microbial perturbation resilience can extend beyond the stressor they originally were exposed to, indicating that ecological memory transcends the original stressor. Repeated perturbation cycles did not confer additional resilience beyond a single event, indicating that a single perturbation could shape the microbial community's perturbation resilience. We also identified the lag phase as a critical period defining microbial perturbation resilience. Our findings demonstrate a broader adaptive capability within microbial communities under climate change so far overlooked, where winter frost could impact summer drought resilience and vice versa, creating a need to consider selective environmental drivers across seasons.

## INTRODUCTION

Abrupt changes in the environment that alter the biomass or composition of ecological communities—pulse disturbances—are ubiquitous in all ecosystems, and are recognized as a decisive aspect of community organization (Jentsch & White, 2019; Levin & Paine, 1974; Menge & Sutherland, 1987). Climate change is predicted to increase the frequency and intensity of pulse disturbances, as the frequency of weather extremes that give rise to heatwaves, storms, droughts, and floods will increase in a warming climate (IPCC, 2021). The urgency of these environmental problems has motivated a concentrated effort to better understand the combined effect of multiple stressors that can each induce pulse disturbances (Hillebrand & Kunze, 2020) across marine (Glibert et al., 2023), freshwater (Vinebrooke et al., 2004), and terrestrial ecosystems (Jentsch & White, 2019).

One critical feedback to Earth's biogeochemical cycles that also underpins essential ecosystem functions and services is the functioning of decomposer microorganisms (Falkowski et al., 2008). Understanding how microbial ecology responds to climate change is therefore crucial for predicting the consequences of the microbially mediated ecosystem–climate feedback in the Anthropocene (Bardgett et al., 2008; Bradford et al., 2016; Hutchins et al., 2019). In land ecosystems, climate change is predicted to increase the frequency and intensity of extreme weather events, particularly perturbations long recognized to be some of the most disruptive for microorganisms in soil, such as alternating cycles of drought–rainfall, as well as freeze–thaw (Harris et al., 2018; IPCC, 2021; Reichstein et al., 2013). These perturbations impose profound stress on microbial communities, repeatedly disrupting their structure and function, with consequences for organic matter decomposition and formation processes, as well as for ecosystem‐level greenhouse gas emissions (Cordero et al., 2023; Jarvis et al., 2007; Rousk & Brangarí, 2022; Schimel & Clein, 1996). The resilience of microbial communities (i.e., their ability to recover metabolism after perturbations) is therefore crucial for maintaining ecosystem stability in the face of escalating climatic variability.

Microbial resilience to perturbation events can be strengthened by repeated pre‐exposure to that same perturbations (Lí, Hicks, Brangarí, & Rousk, 2024; Lí, Hicks, Brangarí, Tájmel, et al., 2024; Meisner et al., 2021; Müller & Bahn, 2022; Rousk & Brangarí, 2022). The material (e.g., physicochemical characteristics) or informational (e.g., species‐associated trait distribution of the community) legacies of past experiences may lead to the formation of an “ecological memory,” which can influence the ability of communities to cope with subsequent perturbations (Brangarí et al., 2021; Canarini et al., 2021; Johnstone et al., 2016). For instance, in soil, repeated exposure to drought cycles has been shown to induce microbial community shifts that confer a higher resilience, as evidenced by a faster recovery of growth rates and higher carbon use efficiencies (CUE) when exposed to new drought cycle events (Brangarí et al., 2021; Hicks et al., 2022). Similarly, a recent study demonstrated that increased exposure to frost cycles favored microbial trait compositions that enhanced the stability and resilience of microbial growth and CUE against subsequent freeze–thaw perturbations (Lí, Hicks, Brangarí, & Rousk, 2024; Lí, Hicks, Brangarí, Tájmel, et al., 2024).

Recent studies suggest that microbial communities experience similar environmental pressures during both drought and frost cycles, largely as a result of the similar fluctuations in soil water availability (Li et al., 2023; Meisner et al., 2021). Both perturbations reduce liquid water content—whether through soil drying or freezing temperatures—and thus cause a drop in water potential. When soils are rewetted by rainfall or when ice thaws as temperatures rise, water availability again increases, allowing the microbial community and its functions to recover. The similar impacts of these two perturbation types raise a question: Can exposure to frost enhance microbial resilience to drought, and vice versa? More generally, can exposure to one type of environmental stress confer microbial resilience or co‐tolerance to another environmental stressor (here termed “cross‐stressor resilience”)? If so, this would recast our understanding of how the environment can filter microbial traits. Addressing this is essential for predicting how microbial processes might adapt to and recover from multiple, cyclic environmental stresses, such as those that occur seasonally in natural environments, thereby influencing ecosystem functional stability to climate change.

While this topic has received substantial attention in aquatic ecosystems, that has amassed numerous studies and meta‐analyses where general patterns have been identified (e.g., Burgess et al., 2021; Hillebrand & Kunze, 2020), it has a much shorter history in soil systems, and is usually limited to either only resolving perturbation resistance (e.g., Rillig et al., 2023) or only including categorical assessments of perturbation recovery (Canarini et al., 2021; Knight et al., 2024; Meisner et al., 2021). Specifically, to date, there are no time‐resolved assessments of microbial cross‐stressor resilience to the key and seasonally interlinked pulse perturbations of drought and frost in soils. In this study, we fill this gap by experimentally testing the microbial resilience to frost and drought cycles and their interaction, by determining the microbial resilience of metabolism to subsequent frost and drought perturbations in two soils exhibiting contrasting microbial resilience to perturbations.

## MATERIALS AND METHODS

### Site description, soil sampling, and experiment preparation

Soils were collected from a natural reserve in Knivsås–Borelund, southern Sweden (55°40′ N, 13°24′ E; 81 m above sea level [asl]), characterized by a temperate oceanic climate, with mean annual precipitation of 680 mm and mean annual temperature of 8°C.

In late November 2020, prior to the onset of frost, soil was sampled from three randomly selected replicate plots (1 m^2^ and >50 m apart) in an oak meadow and a beech forest (i.e., *n* = 3), approximately 5 km apart. Within each plot, soil was sampled from three to five pits down to a depth of 10 cm, combined into plot‐level composite samples, sieved (<4 mm), and immediately transported to the laboratory to initiate the experiments (within 1 week).

**FIGURE 1:**
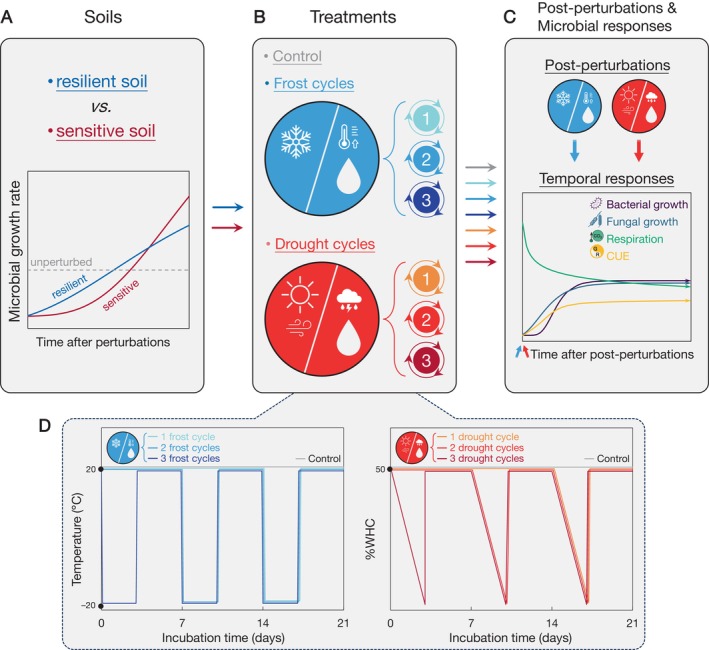
Experimental design. Two contrasting soil types (resilient and sensitive soils; A) were selected and prior exposed to varying frequencies of frost and drought cycles, along with a control treatment, over a 21‐day incubation period (B, D). The temporal responses of soil microbial traits, including bacterial growth, fungal growth, respiration, and carbon use efficiency (CUE), were subsequently examined following an additional cycle of frost or drought perturbation (“post‐perturbation”) (C). All treatments were performed in triplicate (*n* = 3).

Soils from the two selected sites have been previously characterized as exhibiting categorically different microbial growth responses to both drought‐ and frost‐cycle perturbations, despite coming from the same geographical location and thus exposed to the same climate (Li et al., 2023; Winterfeldt et al., 2024). The oak meadow soil (hereafter “resilient soil”) has demonstrated a resilient response, where microbial growth rates start increasing linearly immediately after rewetting or thawing (Figure 1A). In contrast, the beech forest soil (hereafter “sensitive soil”) has shown a more sensitive response, with microbial growth starting only after a lag period of several hours with no apparent growth (Figure 1A). There were significant differences in physicochemical properties of the two soils. Compared to the sensitive soil, the resilient soil has lower soil carbon (C) and nitrogen (N) contents, and a lower C:N ratio (25.6 vs. 95.8 mg C g^−1^ soil, 2.1 vs. 6.1 mg N g^−1^ soil, and 12.3 vs. 15.3, respectively), but higher pH (5.7 vs. 4.2) and NH_4_‐N content (0.14 vs. 0.07 mg N g^−1^ SOM).

After sampling, the moisture content of all soils was first adjusted to 50% water holding capacity (WHC) in 1 L lidded plastic microcosms, which corresponds to a water potential optimal for microbial activity of approximately −30 kPa (Falloon et al., 2011; Schimel, 2018). Subsamples of 1.0 g moist soil were then aliquoted into tubes before being exposed to different treatments, including cycles of drying‐rewetting (drought treatment) and cycles of freezing–thawing (frost treatment).

### Experimental design

The experimental setup comprised two parts. In the first experimental phase (“pretreatments”) (Figure 1B, C), soils were subjected to varying frequencies of drought and frost perturbations over a 3‐week period, consisting of one, two, or three drought or frost cycles (Figure 1D). During this phase, a control group of soils was maintained at 50% WHC and 20°C. In the second experimental phase, soils from the seven pre‐treatments underwent a post‐perturbation consisting of either one additional cycle of drought or frost (Figure 1C).

For each drought cycle, the soils were air‐dried for 3 days at 20°C under a fan, reaching a constant water content of <2% WHC (equivalent to a water potential of approximately −200 MPa), before being rewetted to 50% WHC with distilled water and incubated at 20°C for 4 days. For each frost cycle, the soils were frozen for 3 days at −20°C, followed by a thaw period of 4 days at 20°C. Independent field replicates were maintained throughout all experiments (*n* = 3).

At the end of the first experimental phase, soils from each treatment group were sampled for phospholipid fatty acid (PLFA) analysis to determine microbial biomass and community structure. For the post‐perturbation phase, the moist control group from the pre‐treatment was divided into two subgroups. One subgroup was subjected to a frost or drought perturbation, serving as the “post‐perturbed control” group. The other subgroup was maintained at 50% WHC and 20°C throughout the whole post‐perturbation incubation, serving as the “unperturbed control” group. This unperturbed control provides a stable baseline for microbial dynamics without any additional perturbation, facilitating direct comparison and assessment of treatment legacies on microbial responses upon post‐thawing and rewetting perturbations. After the post‐perturbation, bacterial growth, fungal growth, and respiration rates in all pre‐treated and unperturbed control soils were measured at high temporal resolution through destructive sampling with 13 time points spanning 1 week to capture the dynamic microbial responses (Figure 1C).

### Microbial analyses

Microbial PLFAs were extracted from freeze‐dried subsamples using the method described by Frostegård et al. (1993) with modifications (Cruz‐Paredes et al., 2017). The derived fatty acid methyl esters were quantified on a gas chromatograph with a flame ionization detector. Specific PLFA biomarkers were used to estimate bacterial biomass (i14:0, i15:0, a15:0, i16:0, 16:1ω9, 16:1ω7c, 10Me16:0, i17:0, a17:0, 17:1ω8, cy17:0, 10Me17:0, 18:1ω7, 10Me18:0 and cy19:0), fungal biomass (18:2ω6,9), gram‐positive bacteria (i14:0, i15:0, a15:0, i16:0, i17:0, and a17:0), gram‐negative bacteria (16:1ω9, 16:1ω7, cy17:0, 18:1ω7, and cy19:0), and actinobacteria (10Me17:0, 10Me18:0) (Frostegård & Bååth, 1996; Willers et al., 2015). The combined sum of bacterial, fungal, and nonspecific PLFAs (29 in total) represented total microbial biomass.

Bacterial growth rates were measured by the incorporation of ^3^H‐leucine (Leu) into extracted bacteria (Bååth et al., 2001). Briefly, 1.0 g soil subsamples were mixed with deionized water, centrifuged, and then incubated with 2 μL 1‐[4,5‐^3^H]‐Leucine (5.7 TBq mmol^−1^, Perkin Elmer) and unlabeled Leu at 275 nM. After a 1‐h incubation at 20°C, growth was terminated, and the incorporated radioactivity was measured using a liquid scintillation counter. In parallel, thymidine incorporation was also assessed to enable conversion of bacterial growth rates to carbon units (in micrograms of bacterial carbon production per gram per hour) (Soares & Rousk, 2019). Fungal growth rates were measured by using the acetate‐in‐ergosterol method (Bååth, 2001; Rousk et al., 2009). Briefly, 1.0 g soil samples were mixed with 20 μL of ^14^C‐acetate solution ([1‐^14^C] acetic acid, sodium salt, 2.07 GBq mmol^−1^, Perkin Elmer) and unlabeled sodium acetate, resulting in a final acetate concentration of 220 μM. Samples were then incubated for 2 h at 20°C before formalin was added to terminate growth. The ergosterol was then extracted, separated, and quantified using high‐performance liquid chromatography, and the incorporated radioactivity in the collected ergosterol fraction was determined (Rousk & Bååth, 2007). Fungal growth rates were converted to carbon units (micrograms of fungal carbon production per gram per hour) (Soares & Rousk, 2019). Total microbial growth was then calculated by summing the rates of bacterial and fungal growth, both expressed in carbon units.

Soil respiration was measured by determining the accumulation of CO_2_ in 20 mL headspace vials, which were first purged with pressurized air and sealed with crimp caps. Incubation times varied between 2 and 24 h (with shorter incubation times immediately after the perturbation) such that CO_2_ concentrations in vials allowed both accurate determination without reaching low O_2_ concentrations or excessive CO_2_ concentrations. The CO_2_ concentration was determined using a gas chromatograph (Agilent 8860) equipped with a headspace autosampler (Agilent 7697A) and a thermal conductivity detector.

### Data analysis

#### Characterizing microbial growth

The lag time and recovery time of microbial growth can describe the adaptation and resilience of microbial communities to environmental stress (Allison & Martiny, 2008; Bertrand, 2019; Fridman et al., 2014; Shade et al., 2012). To estimate the lag time and recovery time of microbial growth, we first normalized growth rates to the values measured in unperturbed control soils. For resilient growth responses, where there was an immediate increase in growth rate after the perturbation, the lag time was designated as 0 h.

For sensitive growth responses, where there was an initial lag phase, sigmoid growth models were used to characterize the growth kinetics over time. To avoid the potential assessment bias from the reliance on a single model (Munafò & Davey Smith, 2018), we applied three common empirical models to fit the normalized growth rate (𝑦) against time (𝑡) after post‐perturbations (Baty & Delignette‐Muller, 2004), including a modified Gompertz model (Gibson et al., 1988), the Baranyi model (Baranyi & Roberts, 1994), and the Lag‐exponential model (Buchanan et al., 1997):

Modified Gompertz model:

(1) $$y\left(t\right)=y_{0}+\left(y_{m}-y_{0}\right)\;\exp\left(-\exp\left(1+e\;u_{m}\frac{\lambda -t}{y_{m}-y_{0}}\right)\right).$$

Baranyi model:

(2) $$y\left(t\right)=y_{m}+\ln\left(\frac{\exp\left(\mu_{m}\;t\right)+\exp\left(\mu_{m}\;\lambda \right)-1}{\exp\left(\mu_{m}\;t\right)+\exp\left(\mu_{m}\;\lambda +y_{m}-y_{0}\right)-1}\right).$$

Lag‐exponential model:

(3) $$\begin{array}{c} y\left(t\right)=\left\{\begin{array}{cc} \;y_{0}, & x\leq \lambda \\ y_{m}+u_{m}\left(t-\lambda \right)-\ln\left(\exp\left(y_{m}-y_{0}\right)+\exp\left(u_{m}\left(t-\lambda \right)\right)-1\right), & x>\lambda \end{array}\right. \end{array}.$$

These models share four parameters of biological significance: 𝑦_0_ is the initial rate (lower asymptote), 𝑦_𝑚_ is the maximum rate (upper asymptote), 𝑢_𝑚_ is the maximum specific growth rate, representing the slope at the inflection point, and 𝜆 is the lag time. The recovery time was determined as the time at which the growth equaled 50% of the unperturbed control level, using a numerical approximation method, using Origin 2022 (OriginLab). The 50% recovery level was selected because not all treatments fully recovered, and this level ensures that comparable representations for all treatments could be included in our analysis without missing data. The recovery time to 50% should thus be interpreted as an index useful for comparative analysis (analogous to an effective concentration 50%, “EC50,” value as used in toxicology), where short times indicate a faster recovery than do long times.

Employing multiple models enabled us to validate the robustness of our results and reduce the uncertainty in the interpretation (Munafò & Davey Smith, 2018). We found that the lag or recovery time values obtained from the three models were similar (Appendix S1: Figure S1), indicating the consistency and reliability of each model. Consequently, we report the mean of these three model estimates below. For all soils (both resilient and sensitive growth responses), the recovery was estimated as the time for growth to recover to 50% of the baseline level in the unperturbed control soil.

#### Characterizing microbial CUE

Microbial CUE was estimated as the ratio between total microbial growth (the sum of bacterial and fungal growth) to the total microbial C use (total microbial growth and respiration). We also estimated the recovery of microbial CUE following post‐perturbations using the same approach as described for microbial growth.

#### Statistical analyses

We first tested the effect of perturbation type (control, drought, and frost), frequency (0, 1, 2, and 3 cycles), and their interaction on microbial PLFA biomass using two‐way ANOVA. We further assessed potential shifts in microbial community composition using principal components analysis (PCA) based on relative abundances (in mole percent) of PLFAs, and then tested for treatment effects on the first two principal components (PC1 and PC2) using two‐way ANOVA. We used repeated‐measures ANOVA to analyze the effect of treatment type and frequency and their interaction on the temporal response of microbial traits, including respiration and microbial growth rates, as well as CUE, following post‐perturbations. ANOVA was also conducted to test the effects of treatment type and frequency and their interaction on cumulative microbial growth and respiration, as well as the microbial lag and recovery times. To compare the relative influences of treatments, we calculated the effect sizes using partial eta squared (η^2^_p_) through the *effectsize* package (Ben‐Shachar et al., 2020). All statistical analyses were performed using R (version 4.3.1; R Core Team, 2022). The original data and the code used for statistical analysis in this study are publicly accessible in Zenodo at https://doi.org/10.5281/zenodo.14039010 (Lí, Hicks, Brangarí, & Rousk, 2024).

## RESULTS

### Shifts in microbial biomass and community composition

**FIGURE 2:**
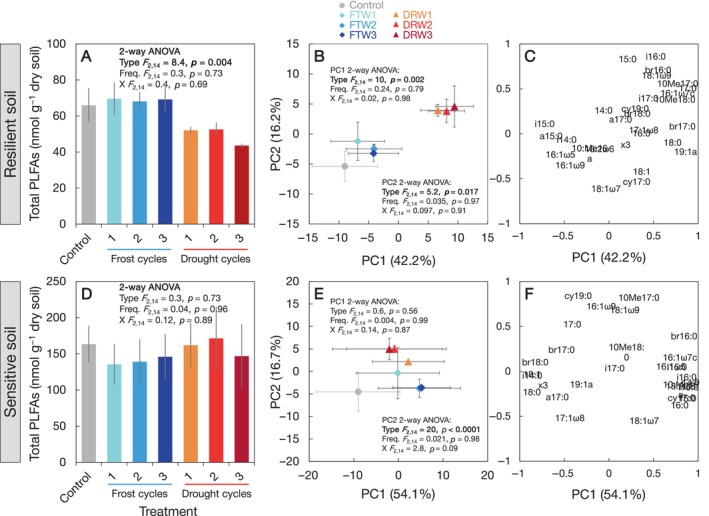
Effects of treatments on soil microbial phospholipid fatty acid (PLFA) concentrations and compositions. The treatments include subjecting soils to varying frequencies (1, 2, and 3 cycles) of frost and drought cycles, along with a control treatment. (A, D) Total microbial PLFA concentrations. (B, E) Treatment scores along the first two principal component (PC) axes. The data indicate the mean values, with error bars representing one SE (*n* = 3). (C, F) Loadings of individual PLFA biomarkers along the first two PC axes. Upper panels (A–C) refer to the resilient soil, while lower panels (D–F) refer to the sensitive soil.

In the resilient soil, total microbial PLFA concentrations were affected by the type of perturbation but not by perturbation frequency (Figure 2A). Drought cycles, but not frost cycles, led to a decrease in total microbial PLFA biomass, with the most pronounced effect in the 3‐drought‐cycle treatment where PLFA biomass was reduced by 34% compared to the control. This pattern was consistent across other group‐specific microbial PLFA biomass (Appendix S1: Figure S2A). Microbial PLFA composition along PC1 and PC2 was affected by the type of perturbation, with drought treatments inducing larger shifts than frost treatments, but not by frequency (Figure 2B,C).

In contrast, the sensitive soil did not exhibit significant changes in total (Figure 2D) or group‐specific (Appendix S1: Figure S2B) PLFA biomass in response to type or frequency of perturbation. However, there was a significant shift in microbial PLFA composition along PC2, which was affected by type of perturbation, but not by frequency, with drought treatments inducing more pronounced shifts in PLFA composition compared to frost treatments (Figure 2E,F).

### Microbial resilience to perturbations

**FIGURE 3:**
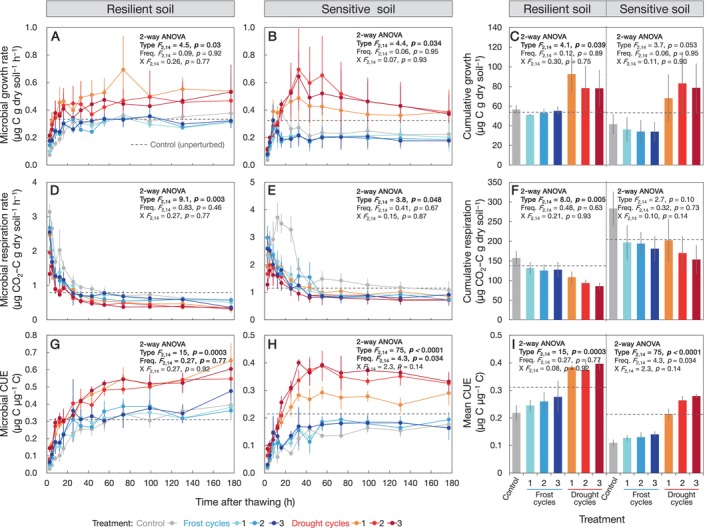
Microbial responses to post‐frost perturbation. The treatments include subjecting soils to varying frequencies (1, 2, and 3 cycles) of frost and drought treatments, along with a control treatment. Temporal dynamics of soil microbial growth rate (A, B), respiration rate (D, E), and carbon use efficiency (CUE; G, H) following thawing frozen soil. Panels on the left (A, D, G) indicate the resilient soil, while panels in the middle (B, E, H) indicate the sensitive soil. The rightmost panels (C, F, I) show cumulative growth (C) and respiration (F), as well as mean CUE (I) over the incubation period in two soil types. The data indicate the mean values, with error bars representing one SE (*n* = 3). The dashed horizontal lines indicate the mean level in control soil that was not exposed to post‐perturbation.

**FIGURE 4:**
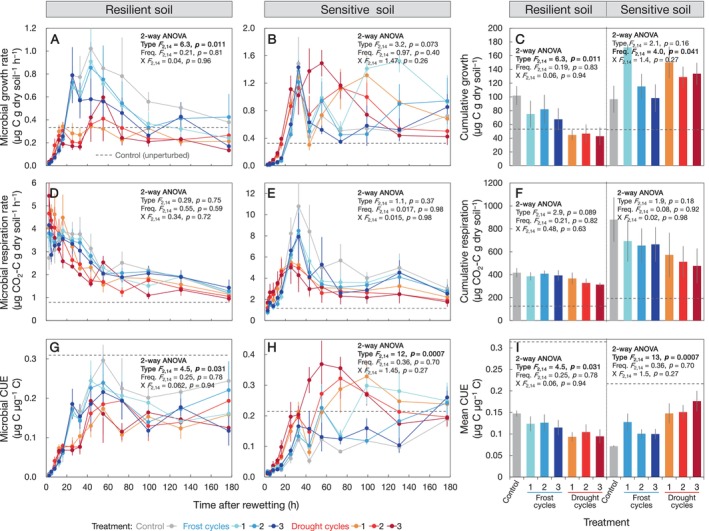
Microbial responses to post‐drought perturbation. The treatments include subjecting soils to varying frequencies (1, 2, and 3 cycles) of frost and drought treatments, along with a control treatment. Temporal dynamics of soil microbial growth rate (A, B), respiration rate (D, E), and carbon use efficiency (CUE; G, H) following rewetting dry soils. Panels on the left (A, D, G) indicate the resilient soil, while panels in the middle (B, E, H) indicate the sensitive soil. The rightmost panels (C, F, I) show cumulative growth (C) and respiration (F), as well as mean CUE (I) over the incubation period. The data indicate the mean values, with error bars representing one SE (*n* = 3). The dashed horizontal lines indicate the mean level in control soil that was not exposed to post‐perturbation.

Following the experimental exposure of the selected sensitive and resilient soils to experimental cycles of frost and drought (Figure 1), the microbial growth and metabolic resilience to both frost (Figure 3) and drought (Figure 4) were comprehensively assessed.

#### Frost resilience

**FIGURE 5:**
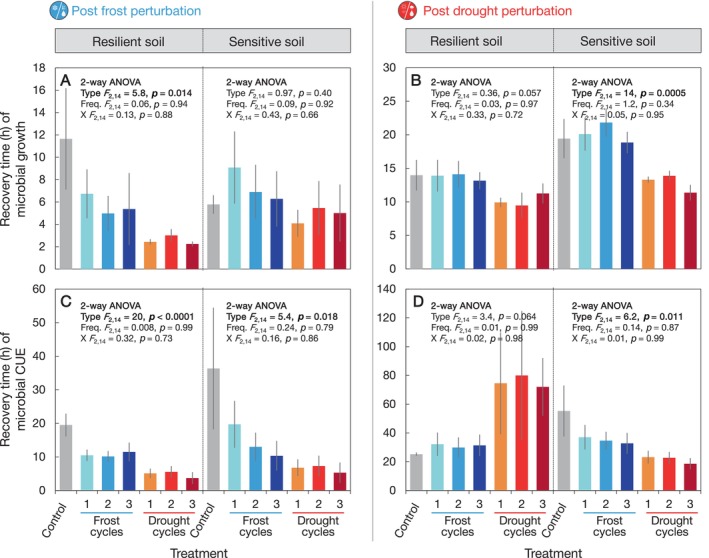
Microbial recovery following post‐frost and drought perturbations. The treatments include subjecting soils to varying frequencies (1, 2, and 3 cycles) of frost and drought treatments, along with a control treatment. The upper panels indicate the recovery time of microbial growth (A, B), while lower panels indicate the recovery time of microbial CUE (C, D) in response to frost (A, C) and drought‐cycle (B, D) perturbations. Recovery time was estimated as the time taken to return to 50% of the baseline level in the unperturbed control soil. The data indicate the mean values, with error bars representing one SE (*n* = 3).

In the resilient soil, microbial growth increased immediately and linearly upon post‐frost perturbation (i.e., thawing frozen soils) across all pre‐treatments (Figure 3A). Microbial growth rates were higher in the drought‐cycle treatments (mean: 0.42 μg C g dry soil^−1^ h^−1^) compared to the control (0.26 μg C g dry soil^−1^ h^−1^) and frost‐cycle treatments (0.27 μg C g dry soil^−1^ h^−1^), with no effect of perturbation frequency (Figure 3A). The recovery of microbial growth after frost perturbation was faster in both the drought‐cycle treatments (~ 3 h to 50% of unperturbed control level) and the frost‐cycle treatments (6 h) than in the control (12 h), with no effect of perturbation frequency (Figure 5A). Microbial growth in both frost‐cycle and control treatments gradually converged with the unperturbed level, whereas microbial growth in drought‐cycle treatments continued to increase, stabilizing at approximately 40% above the unperturbed level (Figure 3A). Microbial respiration peaked immediately after thawing and then rates decreased exponentially to below the unperturbed level across all treatments (Figure 3D). Respiration rates were lower in the drought‐cycle treatments (0.80 μg CO_2_‐C g dry soil^−1^ h^−1^) compared to the frost cycle (1.03 μg CO_2_‐C g dry soil^−1^ h^−1^) and control (1.34 μg CO_2_‐C g dry soil^−1^ h^−1^) treatments, but with no effect of frequency (Figure 3D). Consequently, microbial CUE started increasing immediately and linearly after post‐frost perturbation across all treatments (Figure 3G). The CUE was higher in drought‐cycle treatments (averaging 0.38) compared to control (0.22) and frost‐cycle treatments (0.26), with no effect of frequency (Figure 3G,I). Recovery of microbial CUE after frost disturbance was faster in the drought‐cycle treatments (5 h) and the frost‐cycle treatments (11 h) than in the control (20 h), with no significant effect of frequency (Figure 5C). Microbial CUE in the frost‐cycle and control treatments tended to gradually converge with the unperturbed level, while it continued to rise in the drought‐cycle treatments, stabilizing at 45% above the unperturbed level across three frequency levels (Figure 5G).

A similar pattern of microbial perturbation response was also observed in the sensitive soil (Figure 3). The responses of microbial growth (Figure 3B) and respiration (Figure 3D) were affected by perturbation type but not by frequency. Drought‐cycle treatments resulted in higher growth and generally lower respiration rates compared to control and frost‐cycle treatments (Figure 3B,E). Although microbial growth recovery did not differ among treatments (Figure 5A), microbial growth in the control and frost‐cycle treatments recovered to approximately only 75% and 60% of unperturbed level, respectively, while it recovered to a stable plateau that exceeded the unperturbed level by about 45% in drought‐cycle treatments. Recovery of microbial growth after frost disturbance did not differ significantly between the drought‐cycle (~6 h) and the frost‐cycle treatments (7 h) compared to the control (6 h), with no significant effect of frequency (Figure 5A). Microbial CUE response was affected by both perturbation type and frequency, with perturbation type having a greater effect size (η^2^_p_ = 0.91) compared to frequency (η^2^_p_ = 0.38) (Figure 3H). Higher CUE was observed in drought‐cycle treatments (0.25) than in control (0.11) and frost‐cycle treatments (0.13). Recovery of CUE was faster in both the drought‐cycle treatments (averaging 6 h) and the frost‐cycle treatments (14 h) than in the control (36 h), with no effect of frequency (Figure 5C). Moreover, CUE in control and frost‐cycle treatments recovered to only 70% and 80% of unperturbed level, respectively, while CUE in drought‐cycle treatments stabilized at 55% above unperturbed levels (Figure 3H).

#### Drought resilience

In the resilient soil, microbial growth increased immediately and linearly upon post‐drought perturbation (i.e., rewetting dry soils) across all treatments (Figure 4A). Notably, microbial growth kept increasing to reach a peak before declining in control and frost‐cycle treatments, while drought‐cycle treatments caused a more complex growth response pattern, with an initial peak at 12–16 h and then a secondary, larger peak at 55 h, followed by a decline below the unperturbed levels (Figure 4A). Growth rates were lower in drought‐cycle treatments (0.23 μg C g dry soil^−1^ h^−1^) compared to control (0.47 μg C g dry soil^−1^ h^−1^) and frost‐cycle treatments (0.37 μg C g dry soil^−1^ h^−1^), with no effect of frequency (Figure 4A). Microbial growth recovered faster after drought perturbation in drought‐cycle treatments (~3 h to 50% of unperturbed control level) but not in frost‐cycle treatments (14 h) compared to the control (14 h), with no effect of frequency (Figure 4B). Microbial respiration initially peaked, then decreased to converge with the unperturbed level by the end of the study period across all treatments, with no effect of perturbation type or frequency (Figure 4D). Consequently, microbial CUE started increasing immediately after perturbation across all treatments (Figure 4E). The CUE response was higher in control (averaging 0.15) compared to frost‐cycle (0.12) and drought‐cycle (0.10) treatments (Figure 3E), without effect of frequency (Figure 4G,I). CUE recovery tended to be slower in drought‐cycle (~76 h) and frost‐cycle treatments (31 h) compared to the control (25 h), with no effect of frequency (Figure 4D).

A divergent pattern of microbial perturbation response was observed in the sensitive soil (Figure 4B,E,F). Microbial growth started to increase only after a lag period after rewetting. Growth rate tended to be higher in drought‐cycle treatments (0.61 μg C g dry soil^−1^ h^−1^) compared to control (0.43 μg C g dry soil^−1^ h^−1^) and frost‐cycle treatments (0.51 μg C g dry soil^−1^ h^−1^), with no effect of frequency (Figure 4B). Growth recovery after drought perturbation was faster in drought‐cycle treatments (13 h) than in the control (19 h) but not in frost‐cycle treatments (20 h), with no effect of frequency (Figure 4B). Microbial respiration increased from a low initial value to a peak and then gradually declined (Figure 4E). Respiration peaked immediately after rewetting and, unlike the other soils, continued to increase until it reached its maximum 25–35 h after (Figure 4E). Nevertheless, there was no significant difference in respiration rate (Figure 4E) or cumulation (Figure 4F) among treatments. Microbial CUE started increasing immediately following rewetting across all treatments (Figure 4H). CUE was higher in the drought‐cycle treatments (averaging 0.16) compared to the control (0.07) and frost‐cycle treatments (0.11), with no effect of frequency (Figure 4H,I). CUE recovery after drought disturbance was faster in drought‐cycle treatments (~22 h) and frost‐cycle treatments (35 h) than in the control (55 h), with no effect of frequency (Figure 5D).

### The dependence of microbial perturbation resilience on the lag time

**FIGURE 6:**
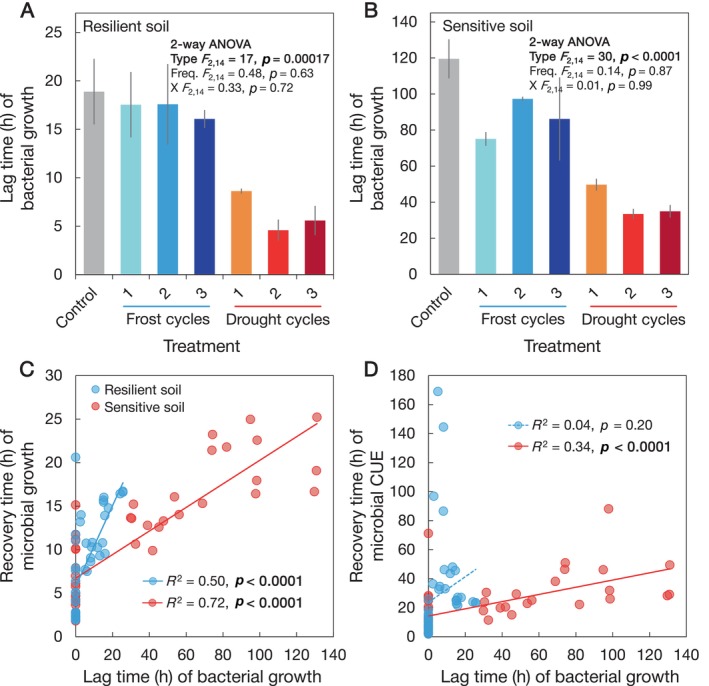
The microbial perturbation resilience and its dependence on lag time. The treatments include subjecting soils to varying frequencies (1, 2, and 3 cycles) of frost and drought treatments, along with a control treatment. (A, B) Lag time of bacterial growth following rewetting dry soil in the resilient (A) and sensitive (B) soils. The data indicate the mean values, with error bars representing one SE (*n* = 3). Note that no detectable lag time was observed upon post‐frost perturbation in either soil type (i.e., lag time = 0 h). (C, D), The linear relationship between lag time and recover times of microbial growth (C) and CUE (D) across both the post‐frost and drought perturbations.

We further investigated whether the recovery of microbial growth and CUE after post‐perturbations were related to the duration of the lag phase across treatments. The results showed consistent treatment effects on the microbial lag time following post‐drought perturbation in both soil types, with shorter lag times in drought‐cycle treatments (mean: 6 h for resilient soil, 39 h for sensitive soil) compared to frost‐cycle (17 and 86 h) and control treatments (19 and 120 h), without effect of frequency (Figure 6A,B). Notably, no detectable lag periods were observed upon post‐frost perturbation in either soil type, indicating a universally immediate response of microbial growth. Furthermore, we found a positive relationship between microbial growth recovery and bacterial lag time across both the post‐frost and drought perturbations in both soil types, with growth recovery more dependent on lag time in resilient soil (slope = 0.46) than in sensitive soil (slope = 0.14) (Figure 6C). Microbial CUE recovery was positively associated with the microbial lag time in the sensitive soil (Figure 6D).

## DISCUSSION

As climate change continues to increase the frequency and intensity of extreme weather events, a general understanding of the microbial ability to recover metabolism after perturbations will be crucial for managing and conserving ecosystem services. This study provides novel insights into the resilience of soil microbial communities to independent environmental perturbations under climate change, specifically examining the effects of prior exposure to drought and frost cycles on subsequent responses to these stressors, and thus the possibility of microbial functions to adapt to extreme perturbation events. Our results demonstrate that microbial communities can develop resilience to a different type of environmental stress than the one they were originally exposed to, where drought exposure can confer improved resilience to frost cycles, and vice versa. The traits that confer resilience to the imposed stressors therefore appear to overlap, resulting in co‐tolerance to the two environmental perturbations. This suggests that ecological memory extends beyond the immediate context of the original stressor. This finding has important implications for understanding how the environment selects on microbial traits, especially within a context where multiple stressors often occur in sequential and seasonally cyclic patterns.

### Perturbation type overrides frequency in shaping microbial resilience

Our results demonstrate that the experimental perturbations (drought and frost) powerfully shaped microbial resilience to subsequent perturbations, indicating that traits that confer resilience to the imposed environmental stressors overlapped, resulting in stressor co‐tolerance (Vinebrooke et al., 2004). Additionally, the stressor co‐tolerance was bidirectional, where drought cycles could confer increased resilience to frost, and vice versa, suggesting that stressor co‐tolerance could be induced independent of the order of stressor exposure, matching both theory and previous empirical interrogations in aquatic systems (MacLennan & Vinebrooke, 2021). The intensity of the experimental stressors differed, however, as evidenced by prior drought‐cycle exposures more strongly enhancing the resilience of microbial growth and CUE to post‐perturbation events, while frost‐cycle exposures had a weaker influence. The difference in intensity of stressors is likely linked to the greater impact of drought‐cycle perturbations on water potential (Ma et al., 2017), thus exerting a stronger selective pressure on microbial traits than frost‐cycle perturbations (Li et al., 2023; Meisner et al., 2021), prompting the development of more robust adaptive strategies to better withstand and recover from subsequent perturbations of this type. Mechanistically, such resilience strategies likely encompass three main pathways: physiological acclimation, community shifts, and evolutionary adaptation (Allison, 2023; Martiny et al., 2015; Schimel, 2018). Physiological acclimation involves rapid changes in gene expression and metabolic pathways that facilitate microbial acclimation upon perturbations (Schimel et al., 2007). Community shifts arise from the selective survival and growth of microbial taxa better adapted to stress, thus leading to a greater relative abundance of species equipped with traits that enable survival and resilience during perturbations (De Vries & Shade, 2013). Evolutionary adaptation is driven by the gradual development of new microbial traits that confer perturbation resilience, allowing communities to endure repeated perturbations over longer timescales (Martiny et al., 2015). The asymmetric strength of the different perturbation types also suggests that, despite likely exerting selective pressure on similar traits linked to coping with variable water potentials, different stressors do not necessarily have arithmetically additive effects. Rather, stressor type dictates the microbial response, with implications for understanding the interactions of multiple compounded stressors associated with climate change.

Interestingly, perturbation frequency had no detectable effect on microbial perturbation resilience, with single and multiple cycles showing similar impacts on microbial recovery of metabolism. This lack of additional resilience benefit arising from repeated perturbation cycles suggests that microbial perturbation resilience was primarily driven by the memory already established (or level of co‐tolerance induced; Vinebrooke et al., 2004) after the initial treatment of perturbation. This finding contrasts with previous studies that suggest that repeated exposure to drought perturbations could further select for more drought‐resilient microorganisms (Evans & Wallenstein, 2012; Fierer & Schimel, 2002), enabling a faster recovery of metabolism to subsequent perturbations (de Nijs et al., 2019; Leizeaga et al., 2022). Our findings are, however, more consistent with the concept of a “priming effect,” in which an initial stimulus might prepare a biological system for future, potentially more severe stressors (Hilker et al., 2016; Rillig et al., 2015) (to be distinguished from the priming effect which refers to short‐term changes in organic matter decomposition in soil science; Kuzyakov et al., 2000). We here found that this “stress priming” may even provide cross‐protection against independent environmental stressors. This finding extends insights from studies showing that perceived predation pressure can prime higher tolerance to high temperatures (MacLennan & Vinebrooke, 2021), that drought‐primed grasses exhibit enhanced frost tolerance (Kreyling et al., 2012), and that exposure to high temperatures primes *Escherichia coli* against low oxygen levels (Tagkopoulos et al., 2008), highlighting that interactions among perturbation factors may well be a rule rather than an exception.

### The role of the lag phase in microbial perturbation resilience

The microbial perturbation resilience is determined by several components, among which is the lag period before growth initiates. We identified a close relationship between the recovery of microbial metabolism indicated by growth and CUE, with the lag duration across both the post‐frost and drought perturbations in both soil types (Figure 6C,D). The lag phase can be explained by the microbial community being so impaired by the perturbation that it requires time to recover their metabolic activity sufficiently to support detectable growth, which suggests that the surviving community was poorly equipped to withstand the perturbation (Brangarí et al., 2021). The lag phase could also enable microbes to undergo essential physiological adjustments critical for their metabolic recovery and adaptation (Bertrand, 2019; Fridman et al., 2014; Shade et al., 2012). For instance, when desiccated microbes are rewetted, they experience rehydration‐related stresses, such as hypoosmotic shock, necessitating metabolic adjustments (Manzoni et al., 2014). To protect against cellular damage, microbes activate several protective systems, including the production of protein‐stabilizing molecules and compatible solutes such as trehalose and glycine betaine, which balance osmotic pressure and protect cellular structures (Potts, 1994). A key aspect of microbial drought and frost resilience in soil thus also lies in their ability to eliminate osmoregulatory compounds when they are no longer needed, restoring osmotic equilibrium once water potential recovers after the perturbation (Brangarí et al., 2021; Warren & Manzoni, 2023). These stress‐adaptation processes can explain the observed lag in microbial responses and highlight the importance of the lag phase as a key period defining microbial resilience to stressors associated with variable water potentials, linked to both frost and drought cycles.

Our results also indicate that this lag phase during which microbes adjust to stress can be shortened by pre‐exposure to the same or independent environmental stressors, accelerating microbial metabolic recovery (Figure 6A,B). A shorter lag phase suggests that drought‐exposed communities had acquired a functional advantage, which could be explained by one or several potential mechanisms. First, physiological acclimation could play a key role, as prior stress exposure may result in changes in gene expression or metabolic pathways that facilitate faster recovery when favorable conditions return (Allison & Martiny, 2008). Second, evolutionary adaptation might contribute, where microbial populations undergo selection over generations for traits that confer increased resilience to recurring stress, promoting metabolic recovery (Shade et al., 2012). Lastly, species sorting and community turnover may also drive this process, as historical stress could lead to the dominance of microbial taxa and emergent trait distributions that are inherently more capable of withstanding and recovering from perturbations (Evans & Wallenstein, 2012).

### Different microbial responses in resilient versus sensitive soils

A striking difference that emerged from our study is that exposure to drought cycles reduced microbial biomass and altered community composition in the resilient soil, while the sensitive soils changed less (Figure 2). This difference may reflect contrasting microbial life history strategies between the two soil types (Malik et al., 2020; Wood et al., 2018). Specifically, in the context of microbial resilience of metabolism and functioning, a reduction in microbial biomass and change in community in the resilient soil may reflect a flexible, short‐term trade‐off strategy, where some populations decline or enter dormancy to conserve resources, allowing for a more effective metabolic recovery of survivor communities later on (Brangarí et al., 2021; De Vries & Shade, 2013). Conversely, the sensitive soil, with less pronounced biomass and community changes, may represent a system already near its functional redundancy and resource allocation limits. While it maintained apparent stability of microbial metabolism, this might reflect a constrained ability to reorganize and recover (Bardgett & Caruso, 2020; Griffiths & Philippot, 2013; Philippot et al., 2021), suggesting that sensitive soils may lack the functional redundancy necessary for long‐term resilience of microbial metabolism and functioning (Bardgett & Caruso, 2020). The contrasting microbial perturbation responses between the two soil types highlight the complexity and variability of microbial dynamics, and the need to integrate soil‐specific factors to improve predictions of microbial resilience to climatic perturbations.

## CONCLUSIONS

We show that induced resilience of soil microbial communities to environmental stressors can extend to independent perturbations. Exposure to drought enhanced microbial functional stability under subsequent exposure to both drought and frost, and exposure to frost enhanced functional stability to subsequent exposure to both frost and drought. This finding extends insights on multiple stressor perturbations from marine and aquatic ecosystems to soil systems, and underscores the importance of considering multiple stressor interactions in predicting future microbial responses to climate change. As such, dry spells in summer can confer improved microbial frost resilience in winter, and vice versa; which is an environmental filtering of microbial traits that so far remains totally overlooked. The type of perturbation exerted a stronger influence on microbial resilience than did their frequency, as repeated cycles of the same perturbation did not improve resilience beyond the first exposure. Overall, our findings provide critical insights into the adaptive capacity of microbial communities amid increasing climate variability, emphasizing the need to explore how such cross‐stressor resilience might affect broader ecosystem functions over the long term.

## AUTHOR CONTRIBUTIONS

**Jin‐Tao Lí:** Data curation; formal analysis; investigation; visualization; writing—original draft. **Lettice C. Hicks:** Investigation; supervision; writing—review and editing. **Albert C. Brangarí:** Investigation; supervision; writing—review and editing. **Johannes Rousk:** Conceptualization; funding acquisition; methodology; project administration; resources; supervision; writing—original draft; writing—review and editing.

## CONFLICT OF INTEREST STATEMENT

The authors declare no conflicts of interest.

## Supporting information


Appendix S1.

## Data Availability

Data and code (Lí, Hicks, Brangarí, & Rousk, 2024) are available in Zenodo at https://doi.org/10.5281/zenodo.14039010.

## References

[ecy70439-bib-0001] Allison, S. D. 2023. “Microbial Drought Resistance May Destabilize Soil Carbon.” Trends in Microbiology 31: 780–787. 37059647 10.1016/j.tim.2023.03.002

[ecy70439-bib-0002] Allison, S. D. , and J. B. H. Martiny . 2008. “Resistance, Resilience, and Redundancy in Microbial Communities.” Proceedings of the National Academy of Sciences 105: 11512–11519. 10.1073/pnas.0801925105 PMC2556421 18695234

[ecy70439-bib-0003] Bååth, E. 2001. “Estimation of Fungal Growth Rates in Soil Using 14C‐Acetate Incorporation Into Ergosterol.” Soil Biology and Biochemistry 33: 2011–2018.

[ecy70439-bib-0004] Bååth, E. , M. Pettersson , and K. H. Söderberg . 2001. “Adaptation of a Rapid and Economical Microcentrifugation Method to Measure Thymidine and Leucine Incorporation by Soil Bacteria.” Soil Biology and Biochemistry 33: 1571–1574.

[ecy70439-bib-0005] Baranyi, J. , and T. A. Roberts . 1994. “A Dynamic Approach to Predicting Bacterial Growth in Food.” International Journal of Food Microbiology 23: 277–294. 7873331 10.1016/0168-1605(94)90157-0

[ecy70439-bib-0006] Bardgett, R. D. , and T. Caruso . 2020. “Soil Mcirobial Community Responses to Climate Extremes: Resistance, Resilience and Transitions to Alternative States.” Philosophical Transactions of the Royal Society B 375: 20190112. 10.1098/rstb.2019.0112 PMC7017770 31983338

[ecy70439-bib-0007] Bardgett, R. D. , C. Freeman , and N. J. Ostle . 2008. “Microbial Contributions to Climate Change through Carbon Cycle Feedbacks.” The ISME Journal 2: 805–814. 18615117 10.1038/ismej.2008.58

[ecy70439-bib-0008] Baty, F. , and M.‐L. Delignette‐Muller . 2004. “Estimating the Bacterial Lag Time: Which Model, which Precision?” International Journal of Food Microbiology 91: 261–277. 14984774 10.1016/j.ijfoodmicro.2003.07.002

[ecy70439-bib-0009] Ben‐Shachar, M. , D. Lüdecke , and D. Makowski . 2020. “Effectsize: Estimation of Effect Size Indices and Standardized Parameters.” The Journal of Open Source Software 5: 2815.

[ecy70439-bib-0010] Bertrand, R. L. 2019. “Lag Phase Is a Dynamic, Organized, Adaptive, and Evolvable Period that Prepares Bacteria for Cell Division.” Journal of Bacteriology 201: 1–21. 10.1128/JB.00697-18 PMC6416914 30642990

[ecy70439-bib-0011] Bradford, M. A. , W. R. Wieder , G. B. Bonan , N. Fierer , P. A. Raymond , and T. W. Crowther . 2016. “Managing Uncertainty in Soil Carbon Feedbacks to Climate Change.” Nature Climate Change 6: 751–758.

[ecy70439-bib-0012] Brangarí, A. C. , S. Manzoni , and J. Rousk . 2021. “The Mechanisms Underpinning Microbial Resilience to Drying and Rewetting – A Model Analysis.” Soil Biology and Biochemistry 162: 108400.

[ecy70439-bib-0013] Buchanan, R. , R. Whiting , and W. Damert . 1997. “When Is Simple Good Enough: A Comparison of the Gompertz, Baranyi, and Three‐Phase Linear Models for Fitting Bacterial Growth Curves.” Food Microbiology 14: 313–326.

[ecy70439-bib-0014] Burgess, B. J. , D. Purves , G. Mace , and D. J. Murrell . 2021. “Classifying Ecosystem Stressor Interactions: Theory Highlights the Data Limitations of the Additive Null Model and the Difficulty in Revealing Ecological Surprises.” Global Change Biology 27: 3052–3065. 33830596 10.1111/gcb.15630

[ecy70439-bib-0015] Canarini, A. , H. Schmidt , L. Fuchslueger , V. Martin , C. W. Herbold , D. Zezula , P. Gündler , et al. 2021. “Ecological Memory of Recurrent Drought Modifies Soil Processes Via Changes in Soil Microbial Community.” Nature Communications 12: 1–14. 10.1038/s41467-021-25675-4 PMC8421443 34489463

[ecy70439-bib-0016] Cordero, I. , A. Leizeaga , L. C. Hicks , J. Rousk , and R. D. Bardgett . 2023. “High Intensity Perturbations Induce an Abrupt Shift in Soil Microbial State.” The ISME Journal 17: 2190–2199. 37814127 10.1038/s41396-023-01512-y PMC10690886

[ecy70439-bib-0017] Cruz‐Paredes, C. , H. Wallander , R. Kjøller , and J. Rousk . 2017. “Using Community Trait‐Distributions to Assign Microbial Responses to pH Changes and Cd in Forest Soils Treated with Wood Ash.” Soil Biology and Biochemistry 112: 153–164.

[ecy70439-bib-0018] de Nijs, E. A. , L. C. Hicks , A. Leizeaga , A. Tietema , and J. Rousk . 2019. “Soil Microbial Moisture Dependences and Responses to Drying–Rewetting: The Legacy of 18 Years Drought.” Global Change Biology 25: 1005–1015. 30387912 10.1111/gcb.14508

[ecy70439-bib-0019] De Vries, F. T. , and A. Shade . 2013. “Controls on Soil Microbial Community Stability under Climate Change.” Frontiers in Microbiology 4: 1–16. 24032030 10.3389/fmicb.2013.00265 PMC3768296

[ecy70439-bib-0020] Evans, S. E. , and M. D. Wallenstein . 2012. “Soil Microbial Community Response to Drying and Rewetting Stress: Does Historical Precipitation Regime Matter?” Biogeochemistry 109: 101–116.

[ecy70439-bib-0021] Falkowski, P. G. , T. Fenchel , and E. F. Delong . 2008. “The Microbial Engines that Drive Earth's Biogeochemical Cycles.” Science 320: 1034–1039. 18497287 10.1126/science.1153213

[ecy70439-bib-0022] Falloon, P. , C. D. Jones , M. Ades , and K. Paul . 2011. “Direct Soil Moisture Controls of Future Global Soil Carbon Changes: An Important Source of Uncertainty.” Global Biogeochemical Cycles 25: 1–14.

[ecy70439-bib-0023] Fierer, N. , and J. P. Schimel . 2002. “Effects of Drying–Rewetting Frequency on Soil Carbon and Nitrogen Transformations.” Soil Biology and Biochemistry 34: 777–787.

[ecy70439-bib-0024] Fridman, O. , A. Goldberg , I. Ronin , N. Shoresh , and N. Q. Balaban . 2014. “Optimization of Lag Time Underlies Antibiotic Tolerance in Evolved Bacterial Populations.” Nature 513: 418–421. 25043002 10.1038/nature13469

[ecy70439-bib-0025] Frostegård, A. , and E. Bååth . 1996. “The Use of Phospholipid Fatty Acid Analysis to Estimate Bacterial and Fungal Biomass in Soil.” Biology and Fertility of Soils 22: 59–65.

[ecy70439-bib-0026] Frostegård, Å. , E. Bååth , and A. Tunlio . 1993. “Shifts in the Structure of Soil Microbial Communities in Limed Forests as Revealed by Phospholipid Fatty Acid Analysis.” Soil Biology and Biochemistry 25: 723–730.

[ecy70439-bib-0027] Gibson, A. M. , N. Bratchell , and T. A. Roberts . 1988. “Predicting Microbial Growth: Growth Responses of Salmonellae in a Laboratory Medium as Affected by pH, Sodium Chloride and Storage Temperature.” International Journal of Food Microbiology 6: 155–178. 3275296 10.1016/0168-1605(88)90051-7

[ecy70439-bib-0028] Glibert, P. M. , W.‐J. Cai , E. R. Hall , M. Li , K. L. Main , K. A. Rose , J. M. Testa , and N. K. Vidyarathna . 2023. “Stressing over the Complexities of Multiple Stressors in Marine and Estuarine Systems.” Ocean‐Land‐Atmosphere Research 2022: 1–27.

[ecy70439-bib-0029] Griffiths, B. S. , and L. Philippot . 2013. “Insights into the Resistance and Resilience of the Soil Microbial Community.” FEMS Microbiology Reviews 37: 112–129. 22568555 10.1111/j.1574-6976.2012.00343.x

[ecy70439-bib-0030] Harris, R. M. B. B. , L. J. Beaumont , T. R. Vance , C. R. Tozer , T. A. Remenyi , S. E. Perkins‐Kirkpatrick , et al. 2018. “Biological Responses to the Press and Pulse of Climate Trends and Extreme Events.” Nature Climate Change 8: 579–587.

[ecy70439-bib-0031] Hicks, L. C. , S. Lin , and J. Rousk . 2022. “Microbial Resilience to Drying‐Rewetting Is Partly Driven by Selection for Quick Colonizers.” Soil Biology and Biochemistry 167: 108581.

[ecy70439-bib-0032] Hilker, M. , J. Schwachtje , M. Baier , S. Balazadeh , I. Bäurle , S. Geiselhardt , D. K. Hincha , et al. 2016. “Priming and Memory of Stress Responses in Organisms Lacking a Nervous System.” Biological Reviews 91: 1118–1133. 26289992 10.1111/brv.12215

[ecy70439-bib-0033] Hillebrand, H. , and C. Kunze . 2020. “Meta‐Analysis on Pulse Disturbances Reveals Differences in Functional and Compositional Recovery across Ecosystems.” Ecology Letters 23: 575–585. 31943698 10.1111/ele.13457

[ecy70439-bib-0034] Hutchins, D. A. , J. K. Jansson , J. V. Remais , V. I. Rich , B. K. Singh , and P. Trivedi . 2019. “Climate Change Microbiology—Problems and Perspectives.” Nature Reviews. Microbiology 17: 391–396. 31092905 10.1038/s41579-019-0178-5

[ecy70439-bib-0035] IPCC . 2021. “Climate Change 2021: The Physical Science Basis ‐ Summary for the Policymakers (Working Group I).” Clim. Chang. 2021 Phys. Sci. Basis.

[ecy70439-bib-0036] Jarvis, P. , A. Rey , C. Petsikos , L. Wingate , M. Rayment , J. Pereira , J. Banza , et al. 2007. “Drying and Wetting of Mediterranean Soils Stimulates Decomposition and Carbon Dioxide Emission: The ‘Birch Effect’.” Tree Physiology 27: 929–940. 17403645 10.1093/treephys/27.7.929

[ecy70439-bib-0037] Jentsch, A. , and D. White . 2019. “A Theory of Pulse Dynamics and Disturbance in Ecology.” Ecology 100: e02734. 31018013 10.1002/ecy.2734 PMC6851700

[ecy70439-bib-0038] Johnstone, J. F. , C. D. Allen , J. F. Franklin , L. E. Frelich , B. J. Harvey , P. E. Higuera , M. C. Mack , et al. 2016. “Changing Disturbance Regimes, Ecological Memory, and Forest Resilience.” Frontiers in Ecology and the Environment 14: 369–378.

[ecy70439-bib-0073] Knight, C. G. , O. Nicolitch , R. I. Griffiths , T. Goodall , B. Jone , C. Weser , H. Langridge , et al. 2024. “Soil Microbiomes Show Consistent and Predictable Responses to Extreme Events.” Nature 636: 690–696. 10.1038/s41586-024-08185-3. 39604724 PMC11655354

[ecy70439-bib-0039] Kreyling, J. , D. Thiel , K. Simmnacher , E. Willner , A. Jentsch , and C. Beierkuhnlein . 2012. “Geographic Origin and Past Climatic Experience Influence the Response to Late Spring Frost in Four Common Grass Species in Central Europe.” Ecography (Cop.) 35: 268–275.

[ecy70439-bib-0040] Kuzyakov, Y. , J. K. Friedel , and K. Stahr . 2000. “Review of Mechanisms and Quantification of Priming Effects.” Soil Biology and Biochemistry 32: 1485–1498.

[ecy70439-bib-0041] Leizeaga, A. , A. Meisner , J. Rousk , and E. Bååth . 2022. “Repeated Drying and Rewetting Cycles Accelerate Bacterial Growth Recovery after Rewetting.” Biology and Fertility of Soils 58: 365–374.

[ecy70439-bib-0042] Levin, S. A. , and R. T. Paine . 1974. “Disturbance, Patch Formation, and Community Structure.” Proceedings of the National Academy of Sciences of the United States of America 71: 2744–2747. 4527752 10.1073/pnas.71.7.2744 PMC388546

[ecy70439-bib-0045] Li, J.‐T. , H. Xu , L. C. Hicks , A. C. Brangarí , and J. Rousk . 2023. “Comparing Soil Microbial Responses to Drying‐Rewetting and Freezing‐Thawing Events.” Soil Biology and Biochemistry 178: 108966.

[ecy70439-bib-0043] Lí, J. , L. C. Hicks , A. C. Brangarí , D. Tájmel , C. Cruz‐Paredes , and J. Rousk . 2024. “Subarctic Winter Warming Promotes Soil Microbial Resilience to Freeze–Thaw Cycles and Enhances the Microbial Carbon Use Efficiency.” Global Change Biology 30: 1–15. 10.1111/gcb.17040 38273522

[ecy70439-bib-0044] Lí, J.‐T. , L. Hicks , A. C. Brangarí , and J. Rousk . 2024. “Cross‐Stressor Resilience of Soil Microbial Growth and Carbon Metabolism under Climate Change [Data Set].” Zenodo. 10.5281/zenodo.14039010 42324861

[ecy70439-bib-0046] Ma, T. , C. Wei , X. Xia , J. Zhou , and P. Chen . 2017. “Soil Freezing and Soil Water Retention Characteristics: Connection and Solute Effects.” Journal of Performance of Constructed Facilities 31: 1–8.

[ecy70439-bib-0075] MacLennan, M. M. , and R. D. Vinebrooke . 2021. “Exposure Order Effects of Consecutive Stressors on Communities: The Role of Co‐Tolerance.” Oikos 130: 2111–2121. 10.1111/oik.08884.

[ecy70439-bib-0047] Malik, A. A. , J. B. H. Martiny , E. L. Brodie , A. C. Martiny , K. K. Treseder , and S. D. Allison . 2020. “Defining Trait‐Based Microbial Strategies with Consequences for Soil Carbon Cycling under Climate Change.” The ISME Journal 14: 1–9. 31554911 10.1038/s41396-019-0510-0 PMC6908601

[ecy70439-bib-0048] Manzoni, S. , S. M. Schaeffer , G. Katul , A. Porporato , and J. P. Schimel . 2014. “A Theoretical Analysis of Microbial Eco‐Physiological and Diffusion Limitations to Carbon Cycling in Drying Soils.” Soil Biology and Biochemistry 73: 69–83.

[ecy70439-bib-0049] Martiny, J. B. H. , S. E. Jones , J. T. Lennon , and A. C. Martiny . 2015. “Microbiomes in Light of Traits: A Phylogenetic Perspective.” Science 350: 1–8. 10.1126/science.aac9323 26542581

[ecy70439-bib-0050] Meisner, A. , B. L. Snoek , J. Nesme , E. Dent , S. Jacquiod , A. T. Classen , and A. Priemé . 2021. “Soil Microbial Legacies Differ Following Drying‐Rewetting and Freezing‐Thawing Cycles.” The ISME Journal 15: 1207–1221. 33408369 10.1038/s41396-020-00844-3 PMC8115648

[ecy70439-bib-0051] Menge, B. A. , and J. P. Sutherland . 1987. “Community Regulation: Variation in Distrubance, Competition, and Predation in Relation to Environmental Stress and Recruitment.” The American Naturalist 110: 351–369.

[ecy70439-bib-0052] Müller, L. M. , and M. Bahn . 2022. “Drought Legacies and Ecosystem Responses to Subsequent Drought.” Global Change Biology 28: 5086–5103. 35607942 10.1111/gcb.16270 PMC9542112

[ecy70439-bib-0053] Munafò, M. R. , and G. Davey Smith . 2018. “Robust Research Needs Many Lines of Evidence.” Nature 553: 399–401. 10.1038/d41586-018-01023-3 29368721

[ecy70439-bib-0054] Philippot, L. , B. S. Griffiths , and S. Langenheder . 2021. “Microbial Community Resilience across Ecosystems and Multiple Disturbances.” Microbiology and Molecular Biology Reviews 85: 85. 10.1128/MMBR.00026-20 PMC8139522 33789927

[ecy70439-bib-0055] Potts, M. 1994. “Desiccation Tolerance of Prokaryotes.” Microbiological Reviews 58: 755–805. 7854254 10.1128/mr.58.4.755-805.1994 PMC372989

[ecy70439-bib-0074] R Core Team . 2022. R: A Language and Environment for Statistical Computing (Version 4.2.1). R Foundation for Statistical Computing. https://www.R-project.org/.

[ecy70439-bib-0056] Reichstein, M. , M. Bahn , P. Ciais , D. Frank , M. D. Mahecha , S. I. Seneviratne , J. Zscheischler , et al. 2013. “Climate Extremes and the Carbon Cycle.” Nature 500: 287–295. 23955228 10.1038/nature12350

[ecy70439-bib-0057] Rillig, M. C. , J. Rolff , B. Tietjen , J. Wehner , and D. R. Andrade‐Linares . 2015. “Community Priming—Effects of Sequential Stressors on Microbial Assemblages.” FEMS Microbiology Ecology 91: 1–7. 10.1093/femsec/fiv040 25873462

[ecy70439-bib-0058] Rillig, M. C. , M. G. A. van der Heijden , M. Bedugo , Y. R. Liu , J. Riedo , C. Sanz‐Lazaro , L. Moreno‐Jiménez , R. Romero , L. Tedersoo , and M. Delgado‐Baquierzo . 2023. “Increasing the Number of Stressors Reduces Soil Ecosystem Services Worldwide.” Nature Climate Change 13: 478. 10.1038/s41558-023-01627-2 PMC7614524 37193246

[ecy70439-bib-0059] Rousk, J. , and E. Bååth . 2007. “Fungal and Bacterial Growth in Soil with Plant Materials of Different C/N Ratios.” FEMS Microbiology Ecology 62: 258–267. 17991019 10.1111/j.1574-6941.2007.00398.x

[ecy70439-bib-0060] Rousk, J. , and A. Brangarí . 2022. “Do the Respiration Pulses Induced by Drying–Rewetting Matter for the Soil–Atmosphere Carbon Balance?” Global Change Biology 28: 3486–3488. 35352861 10.1111/gcb.16163 PMC9314038

[ecy70439-bib-0061] Rousk, J. , P. C. Brookes , and E. Bååth . 2009. “Contrasting Soil pH Effects on Fungal and Bacterial Growth Suggest Functional Redundancy in Carbon Mineralization.” Applied and Environmental Microbiology 75: 1589–1596. 19151179 10.1128/AEM.02775-08 PMC2655475

[ecy70439-bib-0062] Schimel, J. 2018. “Life in Dry Soils: Effects of Drought on Soil Microbial Communities and Processes.” Annual Review of Ecology, Evolution, and Systematics 49: 409–432.

[ecy70439-bib-0063] Schimel, J. , T. C. Balser , and M. Wallenstein . 2007. “Microbial Stress‐Response Physiology and Its Implications for Ecosystem Function.” Ecology 88: 1386–1394. 17601131 10.1890/06-0219

[ecy70439-bib-0064] Schimel, J. , and J. Clein . 1996. “Microbial Response to Freeze‐Thaw Cycles in Tundra and Taiga Soils.” Soil Biology and Biochemistry 28: 1061–1066.

[ecy70439-bib-0065] Shade, A. , H. Peter , S. D. Allison , D. L. Baho , M. Berga , H. Bürgmann , D. H. Huber , et al. 2012. “Fundamentals of Microbial Community Resistance and Resilience.” Frontiers in Microbiology 3: 1–19. 23267351 10.3389/fmicb.2012.00417 PMC3525951

[ecy70439-bib-0066] Soares, M. , and J. Rousk . 2019. “Microbial Growth and Carbon Use Efficiency in Soil: Links to Fungal‐Bacterial Dominance, SOC‐quality and stoichiometry.” Soil Biology and Biochemistry 131: 195–205.

[ecy70439-bib-0067] Tagkopoulos, I. , Y.‐C. Liu , and S. Tavazoie . 2008. “Predictive Behavior within Microbial Genetic Networks.” Science 320: 1313–1317. 18467556 10.1126/science.1154456 PMC2931280

[ecy70439-bib-0068] Vinebrooke, R. D. , K. L. Cottingham , J. Norberg , M. Scheffer , S. I. Dodson , S. C. Maberly , and U. Sommer . 2004. “Impacts of Multiple Stressors on Biodiversity and Ecosystem Functioning: The Rolse of Species co‐Tolerance.” Oikos 104: 451–457.

[ecy70439-bib-0069] Warren, C. R. , and S. Manzoni . 2023. “When Dry Soil Is Re‐Wet, Trehalose Is Respired Instead of Supporting Microbial Growth.” Soil Biology and Biochemistry 184: 109121.

[ecy70439-bib-0070] Willers, C. , P. J. Jansen van Rensburg , and S. Claassens . 2015. “Phospholipid Fatty Acid Profiling of Microbial Communities‐A Review of Interpretations and Recent Applications.” Journal of Applied Microbiology 119: 1207–1218. 26184497 10.1111/jam.12902

[ecy70439-bib-0071] Winterfeldt, S. , C. Cruz‐Paredes , J. Rousk , and A. Leizeaga . 2024. “Microbial Resistance and Resilience to Drought across a European Climate Gradient.” Soil Biology and Biochemistry 199: 109574.

[ecy70439-bib-0072] Wood, J. L. , C. Tang , and A. E. Franks . 2018. “Competitive Traits Are more Important than Stress‐Tolerance Traits in a Cadmium‐Contaminated Rhizosphere: A Role for Trait Theory in Microbial Ecology.” Frontiers in Microbiology 9: 1–12. 29483898 10.3389/fmicb.2018.00121 PMC5816036

