# Supplementary material for: Cross‐stressor resilience of soil microbial growth and carbon metabolism under climate change
Source: Ecology. 2026 Jun 21;107(6):e70439. doi: 10.1002/ecy.70439 (PMC13284524; doi:10.1002/ecy.70439)
Supplement: Supplementary file 1 — Appendix S1. [file ECY-107-e70439-s001.pdf]

Appendix S1 for

Cross-stressor resilience of soil microbial growth and carbon metabolism under climate change

Jin-Tao Lí, Lettice C. Hicks, Albert C. Brangarí, Johannes Rousk

Ecology

Appendix S1 includes:

Figure S1

Figure S2

Supplementary Figures

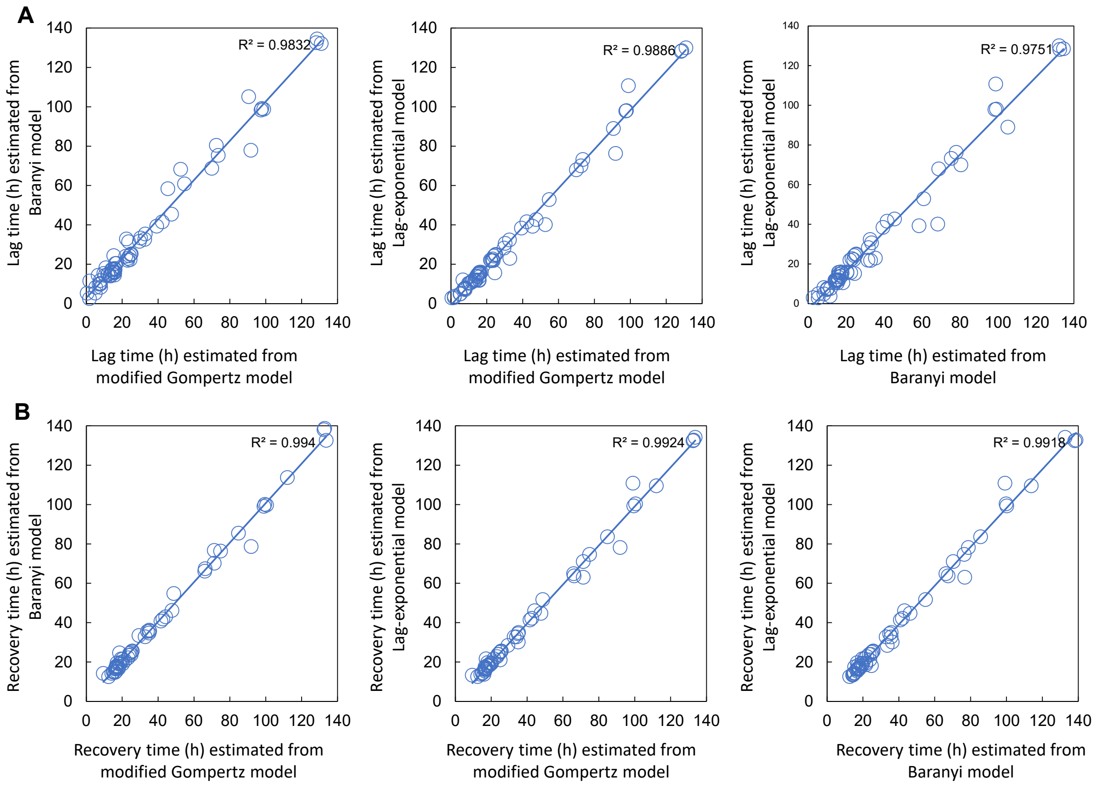

Figure S1. The relationship between lag (A) and recovery (B) times estimated by three sigmoid growth models.

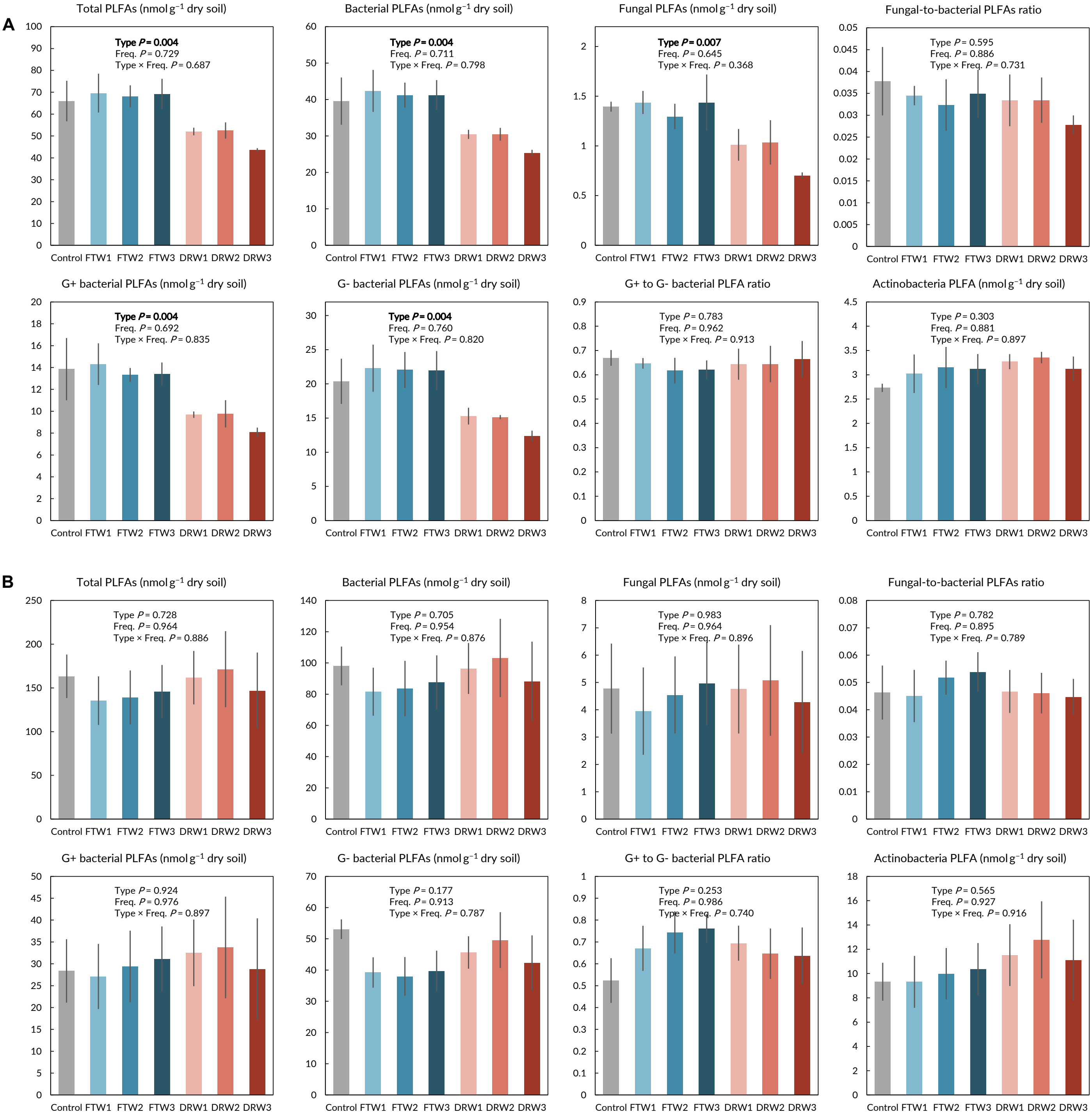

Figure S2. The effect of treatments on microbial PLFA concentrations and ratio.

A, resilient soil; **B**, the sensitive soil.
